# Effects of Chronotype-tailored Bright Light Intervention on Symptoms and Quality of Life in Breast Cancer Survivors

**DOI:** 10.21203/rs.3.rs-3286350/v1

**Published:** 2023-08-25

**Authors:** Horng-Shiuann Wu, Feng Gao, Jean E. Davis, Charles W. Given

**Affiliations:** Michigan State University College of Nursing; Washington University School of Medicine; University of South Carolina College of Nursing; Michigan State University College of Nursing

**Keywords:** bright light, cancer, symptom, polysomnography, quality of life, chronotype

## Abstract

**Purpose:**

Bright light therapy holds promise for reducing common symptoms, e.g., fatigue, experienced by individuals with cancer. This study aimed to examine the effects of a chronotype-tailored bright light intervention on sleep disturbance, fatigue, depressive mood, cognitive dysfunction, and quality of life among post-treatment breast cancer survivors.

**Methods:**

In this two-group randomized controlled trial (NCT03304587), participants were randomized to receive 30-min daily bright blue-green light (12,000 lux) or dim red light (5 lux) either between 19:00–20:00 h or within 30 min of waking in the morning. Self-reported outcomes and in-lab overnight polysomnography sleep study were assessed before (pre-test) and after the 14-day light intervention (post-test).

**Results:**

The sample included 30 women 1–3 years post-completion of chemotherapy and/or radiation for stage I to III breast cancer (mean age = 52.5 ± 8.4 years). There were no significant between-group differences in any of the symptoms or quality of life (all p > 0.05). However, within each group, self-reported sleep disturbance, fatigue, and depressive mood, and quality of life-related functioning showed significant improvements over time (all p < 0.01); the extent of improvement for fatigue and depressive mood was clinically relevant. Polysomnography sleep findings showed that number of awakenings significantly decreased (p = 0.011) among participants received bright light, while stage 2 sleep significantly increased (p = 0.015) among participants received dim-red light.

**Conclusion:**

The findings provide some evidence to support using chronotype-tailored light therapy to manage sleep disturbance, fatigue, depressive mood in post-treatment breast cancer survivors. The unexpected symptom improvements among dim-red light controls remain unexplained and requires further investigation.

**ClinicalTrials.gov Identifier:**

NCT03304587 Study was registered on October 19, 2017.

## INTRODUCTION

Breast cancer survivors currently represent the largest cancer survivor group, comprising more than 3.8 million women in the U.S. [1] and the number of breast cancer survivors continues to grow. Many cancer-related symptoms emerge or amplify during breast cancer treatment and persist long after treatment terminates. A study showed that 88% of breast cancer survivors experienced multiple residual symptoms after treatment completion; half of them experienced six or more concurrent symptoms [2]. Among those, fatigue, sleep disruption, emotional distress, and cognitive dysfunction are most common [3, 4]. These symptoms often exacerbate one another and amplify symptom distress, thus, impeding survivors’ return to a normal and productive life.

Circadian rhythm disruption has been associated with some cancer-related symptoms, e.g., fatigue, sleep disturbance, and depressive mood [5, 6]. Cancer patients suffering greater circadian disruption experienced more disrupted nighttime sleep, more daytime fatigue, greater depression, and worsening life quality [6–8]. Mounting evidence supports bright light’s effect on circadian regulations [9–14]. Bright light therapy has successfully treated circadian rhythm sleep disorders, e.g. shift work and jet-lag, and alleviated fatigue, depression, and insomnia in non-cancerous conditions, e.g., seasonal affective disorder [12, 15–19]. More and more evidence supports the efficacy of bright light therapy in managing symptoms during and after cancer treatment completion [20–31].

In cancer patients, morning bright light has shown the benefit in curbing fatigue, [20, 21, 23, 25–27, 29] but its effects on sleep disturbance were modest [20, 22, 30]. Sleep as measured by actigraphy showed that morning bright light prevented worsening of nighttime sleep disruption and daytime napping during chemotherapy for breast cancer. However, as perceived by individuals, its effect on sleep was suboptimal [31]. A previous study tailored the timing of the light administration according to the individual’s circadian chronotype and showed promise in managing sleep disturbance during chemotherapy [28]. Circadian chronotype (known as morningness-eveningness) is an individual’s natural propensity for sleep/wake timing [12, 32] that stems from the period of endogenous circadian rhythms relative to the 24-hour day/night cycle (circadian phase) [33]. A morningness chronotype demonstrates an earlier diurnal alertness and sleep propensity rhythm (sleep/wake schedule), i.e., tendency of phase advanced from the 24-hour day/night cycle. An eveningness chronotype shows a later sleep/wake schedule, i.e., tendency of circadian phase delay as their circadian period is likely to be longer than 24 hours [34, 35].

Appropriately timed light exposure can augment the effect of bright light [36]. The optimum timing of light exposure is well established [9, 12, 16, 37]. Light exposure in the morning elicits an advance in the time of internal circadian clock relative to external 24-hour clock time. Conversely, light exposure in the later afternoon through early evening delays the circadian rhythm to a later clock time [9, 12, 16, 37]. As an example, for someone who experiences the issue of unintentionally waking up too early (e.g., many older adults), receiving morning light will worsen the problem. It is thought that considering differences in individuals’ chronotypes and customizing the timing of light exposure accordingly can avoid inducing changes in an unwanted direction and worsening already disrupted sleep/wake patterns. Although the chronotype-tailored approach is logically sound, its efficacy is yet to be proven. Thus, the purpose of this study was to estimate the effects of a chronotype-tailored bright light intervention on four symptoms (sleep disturbance, fatigue, depressive mood, cognitive dysfunction) and quality of life among post-treatment breast cancer survivors. Specifically, the hypothesis was: compared to their dim light counterparts, breast cancer survivors who receive bright light intervention would report a significantly greater reduction in sleep disturbance, fatigue, depressive mood, and cognitive dysfunction, and improved quality of life from baseline to post-completion of a 14-day chronotype-tailored light therapy.

## METHODS

### The Chronotype-tailored Intervention Protocol

**Figure 1:**
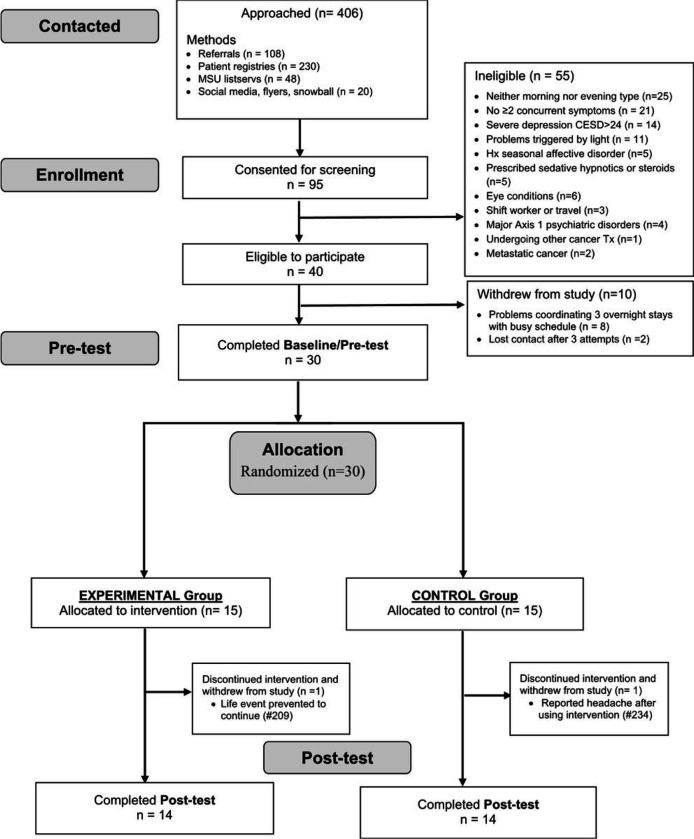
R15 Study Flow Diagram, 11/06/2017 – 8/26/2022

In this two-group randomized controlled trial (NCT03304587) with pre- and post-tests, participants were randomized to either the intervention or control condition using a computer-generated list (Fig. 1). The protocol for both intervention and control groups consisted of a 14-day daily light intervention. Light therapy was self-administered using a light visor cap (Physician Engineered Products, Fryeburg, ME) worn in the individual’s home. Participants in the intervention group self-administered bright blue-green light (~ 500 nm peak; 12,000 lux) once a day for 30 minutes; participants in the control group self-administered dim red light (~ 620nm peak; 5 lux) once a day for 30 minutes. The timing of light administration for both groups was tailored to the individual’s circadian chronotype, based on their natural propensity for sleep/wake time. Chronotype was self-reported based on the Horne-Ostberg Morningness-Eveningness Questionnaire (MEQ) [38]. For evening chronotypes (MEQ scores of ≤ 41), light was delivered within 30 minutes of waking with the goal of advancing circadian phase and, therefore, inducing sleep onset to an earlier time. For morning chronotypes (MEQ ≥ 59), light was delivered in early evening (between 1900–2000 hours) with the goal of delaying circadian phase and, therefore, postponing sleep onset to a later time. Individuals with intermediate types (MEQ scores 42–58) were excluded in this study. Participants were encouraged to use the light therapy at the same time for 30 minutes every day during the study.

Although the light visor contained a timer and automatically turned off after being on for 30 minutes, to promote adherence to the treatment protocol, a multiple-alarm watch with set timed reminders was offered. Most of the participants, however, preferred using the alarm reminder on their own smartphone. The on and off times of each light treatment were self-reported using a daily log to assess adherence.

### Samples and Settings

Participants who resided in the Greater Lansing area in Michigan and within the St. Louis bi-state metropolitan area in Missouri and Illinois were recruited to participate in this three-week long study. Eligible participants were female, 21 years of age or older, 1–3 years post-completion of chemotherapy or/and radiation therapy for stage I-III breast cancer, experience ≥ 2 concurrent symptoms (fatigue, sleep disruption, depressive symptoms, and/or cognitive dysfunction), be either morning or evening chronotypes (MEQ ≥ 59 or ≤ 41), sighted, mentally competent to consent, and able to understand English. Exclusion criteria included a concurrent malignancy; undergoing other cancer treatments; engaged in shift work or traveled across more than three time zones within two weeks prior to the study; a known history of seasonal affective disorder or substance abuse; a current diagnosis of major Axis I psychiatric disorders, neurological impairments, or muscular dystrophies; regular use of steroidal or other immunosuppressive medications; taking prescribed sedative hypnotics or sleep medications; eye conditions (glaucoma or retinal disease) or problems triggered by bright light (e.g., migraine); or taking photosensitizing medications (e.g., some porphyrin drugs, antipsychotics, antiarrhythmic agents). The study was approved by the Institutional Review Boards at Michigan State University in East Lansing, Michigan (IRB #2776) and the Human Investigation Committee at Washington University in St. Louis, Missouri (HRPO#201703147).

### Outcome Variables and Measures

The outcome variables included sleep disturbance, fatigue, depressive mood, cognitive dysfunction, physical function, and quality of life. In addition to subjective report, objective data on sleep disturbance were obtained by in-lab polysomnography (PSG). A description of outcome measures is provided in Table 1.

### Procedure

Potential subjects were recruited via referrals by oncologists or clinic nurses, mail and/or email invitations using patient registries, social media (i.e., Facebook), ResearchMatch.org, and recruitment flyers posted at public areas. The in-person consent/screening visit lasted for one to two hours and was scheduled either on the day of the individual’s clinical appointment or at the individual’s convenience. After giving informed consent, individuals first completed the demographic information followed by the MEQ and four screening instruments with established cut off scores for clinical symptoms, including PSQI, ICD-10 criteria for cancer-related fatigue [39, 40], CES-D, and MoCA. Those who reported the presence of ≥ 2 of the four symptoms were then individually interviewed for the exclusion criteria using a standardized checklist.

After screening, eligible participants were scheduled for the study activities, including three overnight stays at a sleep laboratory. The first overnight stay at the sleep laboratory was an adaptation night. The adaptation night was to facilitate adaptation to sleep study procedures and a new sleep environment and thus controlled for the first night effect. The recorded PSG data during the adaptation night was not analyzed as per standard sleep research methodology.

Baseline data collection occurred on the day following the adaptation night. Prior to checking in to the sleep laboratory, participants were instructed to complete a battery of self-reported instruments. The participants were asked to return to the sleep laboratory around 1900 hours. After checking in, MoCA was administered in person by a trained research assistant. After the completion of the cognition tests, participants were encouraged to relax and engage in their bedtime ritual, e.g., watching TV, reading, etc. The participants were connected to the PSG recording during their normal bedtime hours and underwent overnight PSG monitoring. The recording for the PSG analysis started at the time of lights out and ended at the time of final awakening in the morning.

Starting on the day after the baseline data collection, the participants were instructed to wear the light visor cap at home for 14 consecutive days. A light visor cap and individualized written instructions were provided to the participants before they left the sleep laboratory. Post-test was on the day following the completion of the 14-day intervention protocol using the same protocol procedure as the baseline data collection.

### Data Analysis

The analysis was conducted on an intent-to-treat basis. Demographic and baseline characteristics were tabulated by group and compared using a two-sample t-test, Mann-Whitney rank-sum test, or Chi-square test, as appropriate. The pre- and post-test endpoints in each group were summarized using mean, standard deviation (SD), median, and interquartile range for continuous outcomes, e.g., PROMIS T-scores, or using counts and frequencies for ordinal outcomes, e.g., PSQI component scores. Linear mixed models (for continuous outcomes) or generalized estimating equations (GEE) with cumulative log link function (for ordinal outcomes) were fitted to examine between-group differences, while adjusting for correlation among repeated measures taken from the same participant. Three significance tests were performed simultaneously in each model, including pre-post change in the control group, pre-post change in the experimental group, and the difference in over-time changes between groups. All data analyses were performed using SAS 9.4 (SAS Institutes. Cary, NC) and statistical significance was defined as a two-tailed p-value of < 0.05 for all analyses.

## RESULTS

The data from a convenience sample of 30 female survivors of breast cancer were included in this analysis. Table 2 summarizes the demographics of the study participants. There were no significant differences in individuals’ characteristics between the experimental and control groups.

Two participants (one for each group) reported headaches exaggerated by light. Among those who completed the study (n = 28), counting missed daily records as non-adherence, the intervention vs. control group completed 92% vs. 96% of the planned light treatment sessions. The intervention vs. control group turned on the light visor for an average of 29.78 (± 1.89) vs. 29.73 (± 2.20) minutes per day for an average of 12.9 (± 2.5) vs. 13.4 (± 1.1) days.

### Subjective sleep disturbance.

The symptom scores are listed in Table 3. While between-group differences were not significant, self-reported sleep disturbance significantly decreased in both intervention and control groups after 14 days of light therapy. PROMIS-Sleep Disturbance scores significantly decreased by an average of 6.41 (± 7.31) vs. 6.50 (± 9.61) points in the intervention group vs. control group (with p = 0.009 and p = 0.009, respectively). The reduction in both groups exceeded the pre-set 4.4 MIDs, suggesting the improvements in sleep disturbance are clinically relevant.

Unexpectedly, the PSQI findings favor the control group. Within the control group, PSQI global scores (i.e., overall sleep quality) significantly decreased by 3.50 (± 4.07) points from pre-test to post-test (p = 0.001). In addition, the control group reported significant improvements (lower scores) in five of the seven PSQI sleep components (sleep latency: OR = 0.21, p = 0.002; sleep duration: OR = 0.22, p = 0.002; sleep disturbance: OR = 0.11, p = 0.006; use of medication: OR = 0.44, p = 0.045; daytime dysfunction: OR = 0.18, p = 0.003). Specifically, after receiving 14 days of light therapy, the controls reported significantly shorter sleep onset latency (11.79 ± 14.46 minutes less, p = 0.003) and longer total sleep time (0.49 ± 0.81 more hours, p = 0.029). The intervention group reported significantly lower/improved scores in two of the seven PSQI sleep components, i.e., subjective sleep quality (OR = 0.36, p = 0.031) and sleep latency (OR = 0.34, p = 0.021). However, the pre-post changes in PSQI global score (1.36 ± 2.37, p = 0.140) and sleep onset latency (4.29 ± 12.38 minutes less, p = 0.244) were not statistically significant.

### Objective PSG Findings

The sleep parameters measured by PSG are listed in Table 4. There were no significant between-group differences in any of the PSG sleep parameters (all p > 0.05). Within the intervention group, number of awakenings significantly decreased by an average of 4.82 (± 7.28) awakes from pre-test to post-test (p = 0.011); however, wake after sleep onset (WASO) significantly increased by an average of 21.18 (± 39.35) minutes (p = 0.057). Within the control group, stage 2 sleep significantly increased by 6.20 (± 9.15)% from pre-test to post-test (p = 0.015) (40.87 ± 84.47 minutes, p = 0.056). In addition, although the differences were not statistically significant, the intervention group had shortened sleep latency by 15.67 (± 50.84) minutes while control group had prolonged total sleep time by 22.83 (± 99.04) minutes. However, both groups showed non-significant decreases in stage 3 sleep and rapid eye movement (REM) sleep from pre-test to post-test.

#### Fatigue, depressive mood, and cognitive dysfunction.

Fatigue severity significantly decreased in both intervention and control groups after 14 days of light therapy, but between-group differences were not significant. PROMIS-fatigue scores significantly decreased by an average of 6.96 (± 5.84) vs. 6.89 (± 6.60) points in the intervention group vs. control group (both p < 0.001). The reduction in fatigue in both groups exceeded the pre-set 4.0 MIDs, suggesting the changes are clinically relevant.

Depressive mood significantly declined in both intervention and control groups after 14 days of light therapy, but between-group differences were not significant. PROMIS-Depression scores significantly decreased in both intervention and control groups by an average of 4.57 (± 3.82) and 5.06 (± 6.19) (p = 0.003 and p = 0.001), respectively. The reduction in depressive mood in both groups exceeded the pre-set 4.0 MIDs.

Unexpectedly, neither bright nor dim light condition demonstrated a positive effect on cognitive dysfunction. After adjusting for the baseline MoCA scores, no meaningful changes were observed in either group (intervention: 0.00 ± 0.92, p = 0.99; control: 0.64 ± 2.17, p = 0.251).

### Physical Function and Quality of Life

The quality of life scores are listed in Table 3. After receiving 14 days of light therapy, the intervention group reported significant improvements in global health status / quality of life (QOL) and QOL-related functioning while the control group reported significant improvements in QOL-related symptomology and functioning. QOL-global health status improved in both intervention group (9.52 ± 11.72, p = 0.006) and control group (5.36 ± 12.06, p = 0.104), though the change in the control group was not statistically significant. On the other hand, QOL-related symptomology significantly decreased in the control group (7.69 ± 9.43, p = 0.018) but the reduction in the intervention group was only marginally significant (6.13 ± 13.06, p = 0.051). QOL-related functioning significantly improved in both intervention and control groups by an average of 7.94 (± 9.29) and 8.10 (± 10.55) points (p = 0.006 and p = 0.005), respectively. However, only the control group showed significant improvements in the PROMIS-physical function scores (2.83 ± 6.27, p = 0.035).

## DISCUSSION

The findings from this study did not support our hypothesis that bright blue-green light is superior to dim-red light in reducing self-reported sleep disturbance, fatigue, depressive mood, and cognitive dysfunction, and improving physical function and QOL. Although no significant group effects were displayed by the end of the 14-day light intervention, changes over time were significant within each light condition after adjusting for baseline values. In contrast to our hypothesis, the study results are equally favorable to the dim red-light condition that intended to serve as the control. Those who received dim red-light reported significant improvements in self-reported sleep disturbance, fatigue, depressive mood, physical function, QOL-related symptomology, and QOL-related functioning while those who received bright blue-green light reported significant improvements in fatigue, depressive mood, and QOL-global health status. Both light conditions demonstrated significant and beneficial effects on fatigue and depressive mood. In either light condition, the extent of improvement for fatigue and depressive mood exceeded the pre-selected MIDs and thus was clinically relevant.

Although overall, participants self-reported sleep findings were favorable for both light conditions, PSG findings showed favorable trends in improved total sleep time and WASO among those who received dim light; and, shortened sleep onset latency among those who received bright light. Further, depending on the instrument and sleep parameter studied, some inconsistent self-reported findings were identified. For example, both light conditions showed statistically significant and clinically meaningful improvements in sleep disturbance (measured by PROMIS-Sleep Disturbance). While sleep disturbance (measured by PROMIS-Sleep Disturbance) significantly reduced among those who received bright blue-green light, their overall sleep quality (measured by PSQI global scores) did not improve. Similarly, while physical function (measured by PROMIS-Physical Function) only improved among those who received dim-red light, QOL-related functioning significantly improved in both light conditions. Because the instruments used in this study are all psychometrically sound, the inconsistencies are likely due to instrumentation differences. Whether different sleep parameters (e.g., sleep disturbance vs. sleep onset latency) are affected differently by light requires further investigation. The observed decreases in Stage 3 and REM sleep in both light conditions are yet to be understood.

The findings of dim-red light effects were unexpected. However, similar findings have been reported in existing cancer studies. In the study conducted by Starreveld and colleagues [27], the dim-white light (8 lux) controls reported significant reductions in fatigue and depression and improvements in sleep quality and QOL after receiving 25 days of light therapy. In the study by Johnson and colleagues [23], the dim-red light (< 400 lux) controls reported significant over time improvements in fatigue, mood disturbance, depression, and QOL after 28 days of light therapy. Like our findings, no significant group differences were found. In these two studies, both bright and dim light conditions demonstrated significant over time improvements, with some changes that could be clinically meaningful. Other studies also reported significant improvements in fatigue in their dim-red light controls [25, 26].

Rationale for the improvement observed in dim light controls includes diminished exposure or darkness effects (with light visor caps), social cue and daily routine, response shift, and placebo effects [23, 27, 29]. Dim-red light is often used as the control to overcome placebo effects in studies involving bright light therapy [26, 41] as intrinsically photosensitive retinal ganglion cells (ipRGCs) was thought to be insensitive to long wavelength (red) light [42, 43]. Although it is yet to be proven, it is plausible that exposure to dim red light produces therapeutic effects. It has been suggested that humans may be more sensitive to light than currently known [44]. Relatively dim (as low as 10 lux) light exposure in the evening showed effects on circadian rhythms among healthy adults [45]. To rule out the effect of dim light requires the comparison between two light conditions with either fixed intensity or spectrum/wavelength. However, in this study, two different light spectrums (blue-green: ~500 nm peak vs. red: ~620nm peak) each with different light intensity (bright: 12,000 lux vs. dim: 5 lux) were used. Therefore, we were unable to tease out which elements of light (i.e., spectrum or intensity) contributed to the observed improvements.

Furthermore, different from previous studies in which bright light was uniformly delivered in the morning, our participants received either morning or evening light according to their chronotypes. It is known that the response varies with not only light intensity but also timing of light exposure [9, 12, 16, 37]. The majority (73%) of our participants received evening light because of their morning chronotypes. Although it is conceivable that the timing of light administration coupled with the wavelength enhanced the effects, the explanation of the observed effect of dim light conditions remains open and in need of further research.

Light exposure at the appropriate portion of the phase response curve has been suggested to augment the effect of bright light [36]. Although the observed sleep improvements after the 14 days of chronotype-tailored light therapy in either of our light conditions are compelling, whether the chronotype-tailored approach is superior to morning light remains unanswered. Because of the differences in instruments, patient populations (during chemotherapy vs. post-treatment), light intensities (1,250 to 1,500 lux), intervention duration (25 days to 12 weeks), and other concurrent treatment (cognitive behavioral therapy), [21, 27, 30, 31] comparisons cannot be made across the studies.

The major weaknesses are small sample size and the limited generalizability of the results. Because the samples were all females, post-menopaused, diagnosed with mostly early-stage breast cancer, either morning or evening chronotypes, the findings may not be applicable to males, late-stage breast and/or other cancers, and intermediate chronotypes. In addition, the inclusion criterion was limited to the first three years after completion of chemotherapy and/or radiation, the findings may not be applicable to long-term survivors. Furthermore, the sustainability of the effects of light is unknown.

The findings provide some evidence to support chronotype-tailored light therapy as a promising non-pharmacological intervention that is in-expensive, easy to implement, and relatively safe for managing sleep disturbance, fatigue, depressive mood in post-treatment breast cancer survivors. Unexpectedly, we found that the participants in the control group benefited even more from the dim red light. Some unexpected findings remain unexplained, but nonetheless future research needs to explore the potential of using dim light as an alternative option as the users who cannot tolerate bright light have been precluded. If light is used at the proper time, even with lower intensity, it may promote advantageous changes in sleep/wake patterns and have positive impact on other symptoms, e.g., fatigue and depressive mood, and quality of life.

## Figures and Tables

**Table 1:** Study outcome measures

Variable	Measure	Description of Measure

Sleep Disturbance	Patient-Reported Outcomes Measurement Information System (PROMIS)-Sleep Disturbance Short Form 8a v1.0	• 8 items with 5-point rating scales measuring overall sleep and sleep-related impairments during the past 7 days • Higher T-scores indicate greater sleep disturbance • Validity was supported by moderate to high correlations with the existing scales, e.g., PSQI, Epworth Sleepiness Scale. The scores significantly differed among participants with and without sleep disorders. Minimally important differences (MIDs) of 4.4 for the PROMIS-Sleep Disturbance was previously established in breast cancer patients receiving chemotherapy

	Pittsburgh Sleep Quality Index (PSQI)	• 19 items measuring sleep quality and disturbance in the past month • The 19 items yield seven sleep characteristics/components., i.e., sleep quality, latency, duration, efficiency, disturbance, medication use, and daytime dysfunction. • Each component score is rated on a 0–3 rating scale; the global PSQI score is the accumulative score of seven components, range 0–21, with higher scores indicating more severe sleep disturbance • A global PSQI score greater than 5 was found to have a sensitivity of 89.6% and a specificity of 86.5% in differentiating good and poor sleepers. In a sample of cancer patients, internal consistency reliability was α = 0.81 and 0.69 for the global sleep quality sleep and disturbance subscales, respectively.

	Polysomnography	• 10 mm gold cup disc electroencephalograph (EEG), electromyograph (EMG), and electrooculograph (EOG) electrodes were connected to a Sandman system, version 10.1.3 (Natus, Middleton, WI) • A standard sleep montage for PSG was used. Scalp electrodes were applied following the internationally recognized 10/20 system for electrode placement to record brain waves (electroencephalogram). Eye electrodes were placed one centimeter above or below the outer canthus of the right and left eye to record eye movements (electrooculogram) and one chin electrode were placed on the mental midline to record muscle tone (electromyogram) • Data were visually scored by a Registered Polysomnographic Sleep Technologist blind to the group assignment following the American Academy of Sleep Medicine Manual

Fatigue	PROMIS-Fatigue Short Form 8a v1.0	• 8 items measuring fatigue experience (frequency, duration, and intensity) and fatigue impact (physical, mental, and social activities) during the past 7 days with 5-point rating scales (1 = *not at all or never,* 5 = *very much* or *always*) • Higher T-scores indicate worse fatigue. • Developed based on rigorous methodologies. Psychometric properties have been established across chronic illnesses including cancer • T-scores of 2.5–5.0 for MIDs of the PROMIS- fatigue were previously established in advanced cancer patients; MID of 4.0 was selected as used in an existing study of breast cancer

Depressive Mood	PROMIS-Emotional Distress-Depression Short Form 8a v1.0	• 8 items with 5-point rating scales (1 = *never* to 5 = *always*) measuring affective and cognitive manifestations of depressive mood in the past 7 days • Higher T-scores indicate worse depression • In a sample of depressed outpatients, PROMIS-Depression showed greater reliability when compared to the CES-D and the Patient Health Questionnaire (PHQ-9). Convergent validity with the CES-D and PHQ-9 was supported by strong correlations, ranging from 0.72 to 0.84 • T-scores of 3.0–4.5 for MIDs of the PROMIS-depression were previously established in the oncology population; MID of 4.0 was set as used in an existing study of breast cancer

Cognitive Dysfunction	Montreal Cognitive Assessment (MoCA)	• There are 3 alternate forms designed for use in longitudinal studies • The MoCA is a 30-point scale with seven cognitive subtests: visuo-executive, naming, attention, language, abstraction, delayed recall, and orientation • An extra point is given to a person who has equal or less than 12 years of formal education. The score ranges from 0 to 30, where higher scores indicate better cognition and a score below 26 indicates cognitive impairment. • The MoCA is highly sensitive for screening patients with mild cognitive impairment

Physical Function	PROMIS-Physical Function-Short Form 8b v1.2	• 8 items with 5-point rating scales measuring the individual's ability to complete daily activities • Higher T-scores indicate better functioning • Validity was tested in 1,415 adults with diverse clinical conditions. The scores corresponded to the expected positive or negative changes in the individual's physical function • T-scores of 4.0–6.0 for MIDs of the PROMIS-Physical function was previously established; MIDs of 4.0 was selected as used in an existing study

Quality of Life	European Organization for Research and Treatment of Cancer-Quality of Life Questionnaire (EORTC QLQ-C30)	• 30 items with 4-point rating scales (1 = *not at all*, 4 = *very much*) measuring functioning, symptom intensity, and global health status • Internal consistency α of the functioning and symptoms subscales ranged from 0.54 to 0.86 in lung cancer patients before and during cancer treatments. Known-group comparisons showed differences between patients differing in clinical status

**Table 2:** Characteristics of Participants (N = 30)

	Experimental (n = 15)		Control (n = 15)		

	Mean (SD)	Number (valid %)	Mean (SD)	Number (valid %)	Non-Parametric p-value

**Age** (years)	50.8 (± 8.1)		54.2 (± 8.7)		.46

**Race**		14 (93.3)		14 (93.3)	>0.99
White		1 (6.7)		1 (6.7)	
Black					

**Education**	15.6		16.5		.32
(years)	(± 2.7)		(± 2.6)		

**Marital Status**		1 (6.7)		1 (6.7)	.83
Single		11 (73.3)		9 (60.0)	
Married/Partnered		3 (20.0)		4 (26.7)	
Divorced		0 (0.0)		1 (6.7)	
Widowed					

**Employment**		9 (60.0)		10 (66.7)	.49
Full-time		1 (6.7)		1 (6.7)	
Part-time		4 (26.7)		1 (6.7)	
Self-employed		1 (6.7)		3 (20.0)	
Retired					

**Living Arrangement**		1 (6.7)		2 (13.3)	>0.99
Live alone		14 (93.3)		13 (86.7)	
Live with others					

**Tumor Stage**		8 (53.3)		11 (73.3)	.55
Stage I		5 (33.3)		3 (20.0)	
Stage II		2 (13.3)		1 (6.7)	
Stage III					

**Previous Treatment**		2 (13.3)		1 (6.7)	.76
Chemotherapy		8 (53.3)		10 (66.7)	
Radiation		5 (33.3)		4 (26.7)	
Both					

**Numbers of Comorbidities**		7 (46.7)		6 (42.9)	.91
0		5 (33.3)		3 (21.4)	
1		3 (20.0)		3 (21.4)	
2		0		21 (14.3)	
≥ 3					

**Chronotype**		4 (27.0)		4 (27.0)	1.0
Eveningness		11 (73.0)		11 (73.0)	
Morningness					

**Table 3:** Mean (SD) Symptom and quality of life scores by group condition

	Bright Light		Dim Light	

	Baseline	Post-test	Baseline	Post-test
	(n = 15)	(n = 14)	(n = 15)	(n = 14)

PSQI-	9.3	7.8	9.6	6.0^b^
Global Sleep	(3.2)	(2.4)	(3.2)	(3.1)

Subjective Sleep Quality	1.7	1.4^a^	1.5	1.1
	(0.5)	(0.5)	(0.6)	(0.6)

Sleep Latency	1.6	1.1^a^	1.7	0.8^b^
(Minutes)	(0.9)	(0.8)	(11)	(0.7)
	25.7	21.1	29.8	14.8^b^
	(17.0)	(14.3)	(22.7)	(8.3)

Sleep Duration	1.1	1.1	1.1	0.6^b^
(Hours)	(0.8)	(0.8)	(0.6)	(0.5)
	6.3	6.3	6.5	7.1^a^
	(12)	(11)	(0.9)	(0.7)

Habitual Sleep Efficiency	1.0	1.0	0.9	0.5
(%)	(12)	(12)	(11)	(0.7)
	79.6	80.1	81.9	86.0
	(12.2)	(12.0)	(13.4)	(8.7)

Sleep Disturbance	1.7	1.6	1.9	1.4^b^
	(0.5)	(0.7)	(0.5)	(0.5)

Use of Sleep Medication	1.0	0.5	1.1	0.7^a^
	(12)	(0.9)	(11)	(10)

Daytime Dysfunction	1.3	1.1	1.3	0.9^b^
	(0.7)	(0.5)	(0.9)	(0.8)

PROMIS-Sleep Disturbance	56.0	49.6^b^	57.3	50.4^b^
	(6.0)	(5.3)	(7.5)	(6.0)

PROMIS-Fatigue	55.8	48.3^b^	56.3	50.5^b^
	(6.8)	(4.4)	(7.9)	(6.4)

PROMIS-Depression	50.4	45.2^b^	51.1	46.5^b^
	(7.9)	(5.9)	(6.4)	(7.0)

MoCA-Cognitive Dysfunction	27.9	28.0	26.6	27.1
	(1.7)	(2.0)	(2.2)	(1.7)

PROMIS-Physical Function	47.5	48.7	48.6	51.3^a^
	(10.2)	(9.2)	(8.1)	(8.3)

QOL-Global Health	65.6	76.2^b^	76.1	81.0
	(16.6)	(7.9)	(16.0)	(11.0)

QOL-Symptom	20.7	13.4	19.5	12.1^a^
	(13.0)	(7.1)	(9.0)	(7.3)

QOL-Function	76.7	87.0^b^	80.4	87.6^b^
	(15.7)	(6.6)	(10.4)	(9.2)

Compared to baseline in each group:

a p< .05

b p< .01

**Table 4:** Mean (SD) Polysomnography (PSG) scores by group condition

	Bright ligh		Dim light	

	Baseline	Post-test	Baseline	Post-test
	(n = 15)	(n = 14)	(n = 14)	(n = 14)

Total Sleep Time	394.2	386.1	393.53	415.9
	(68.9)	(84.8)	(59.35)	(71.5)

Sleep Efficiency (%)	81.9	81.0	82.7	82.4
	(7.9)	(14.3)	(8.08)	(12.2)

Sleep Onset Latency (Min)	58.6	35.7	43.9	52.4
	(64.8)	(33.1)	(49.5)	(55.4)

Awakenings	20.5	15.6^a^	16.4	15.2
	(8.6)	(5.2)	(7.1)	(6.0)

Arousals	53.4	45.8	64.6	58.0
	(20.4)	(17.9)	(40.7)	(32.2)

Arousal Index	8.4	7.8	9.6	8.1
	(3.5)	(2.8)	(5.3)	(4.0)

Wake After Sleep Onset	51.6	72.8	47.8	48.2
(WASO)	(19.9)	(41.5)	(22.7)	(31.4)

Stage 1 (Min)	26.0	24.7	28.2	26.1
	(12.6)	(7.9)	(13.9)	(14.5)

Stage 2 (Min)	236.7	250.3	241.2	279.9
	(49.1)	(61.3)	(55.4)	(74.3)

Stage 3 (Min)	37.7	27.1	32.5	23.4
	(40.0)	(30.2)	(23.4)	(27.4)

REM (Min)	93.9	84.0	91.7	86.5
	(30.7)	(28.9)	(28.9)	(33.9)

Stage 1 (%)	6.9	6.7	7.4	6.3
	(4.0)	(2.9)	(4.4)	(3.3)

Stage 2 (%)	60.6	64.8	61.0	66.7^a^
	(10.7)	(7.7)	(7.7)	(11.0)

Stage 3 (%)	9.2	7.3	8.5	6.4
	(9.0)	(7.5)	(6.6)	(8.4)

REM (%)	23.4	21.2	23.2	20.7
	(5.4)	(5.8)	(6.5)	(6.9)

Compared to baseline in each group:

a p< .05

## Data Availability

The data that support the findings of this study are available on request from the corresponding author. The data are not publicly available due to privacy or ethical restrictions.
